# Orthobiologics in knee osteoarthritis, dream or reality?

**DOI:** 10.1007/s00402-024-05310-9

**Published:** 2024-04-17

**Authors:** Nicolaas Cyrillus Budhiparama, Dananjaya Putramega, Imelda Lumban-Gaol

**Affiliations:** 1 Department of Orthopaedics, Leiden University Medical Center, Leiden, The Netherlands; 2 Department of Orthopaedic and Traumatology, Faculty of Medicine, Universitas Airlangga, Jl. Mayjend. Prof. Dr. Moestopo 6–8, Surabaya, 60286 Indonesia; 3 Nicolaas Institute of Constructive Orthopaedic Research & Education Foundation for Arthroplasty & Sports Medicine at Medistra Hospital, Jl. Jend. Gatot Subroto Kav. 59, Jakarta, 12950 Indonesia; 4 Academic Hospital Universitas Gadjah Mada, Yogyakarta, Indonesia

**Keywords:** Orthobiologic, Mesenchymal stem cells, Platelet-rich plasma, Gene therapy, Knee osteoarthritis

## Abstract

Cartilage restoration or repair, also known as orthobiologic therapy, is indicated after the failure of conservative or supportive treatment. However, there is paucity in evidence supporting the efficacy of orthobiologic therapy. The blood-derived products, such as platelet-rich plasma (PRP), is one of the commonly used orthobiologic therapy for knee osteoarthritis. Several studies have shown that PRP is superior to other treatments, but the anatomic changes are scarce. Treatment with mesenchymal stem cells (MSCs) offers the greatest potential for curing degenerative disease due to their self-renewal ability, ability to migrate towards injured tissues (homing/trafficking), and ability to promote repair and regeneration of osteochondral defects. However, ethical concerns and high costs remain major challenges associated with MSC therapy. Gene therapy, another promising orthobiologic therapy, is currently in phase II clinical trial and has shown promising results. The key factors for successful orthobiologic therapy include patient selection, appropriate dosing, treatment of underlying mechanical problems, age, severity, and cost-effectiveness.

Orthobiologic therapy, or “cellular arthroplasty”, involves substances that are autologous in origin and are processed to higher concentrations to speed up and enhance the quality of soft tissue healing. Today, orthobiologics are considered one of the nonoperative modalities for knee osteoarthritis. As part of emerging regenerative medicine, this modality aims to reduce symptoms and disease progression by slowing the degenerative process, accelerating soft tissue regeneration, and promoting anti-inflammatory agents to combat the inflammatory process inside the joint.

Why orthobiologics? Osteoarthritic knees are well known to have a low intrinsic regeneration potential of cartilage, which might be due to the difficulty encountered by progenitor cells from the blood, bone marrow, or even other compartments to enter the defect and the inability of resident articular chondrocytes that are entrapped within the surrounding matrix to migrate into the lesion to secrete a reparative matrix [91]. 

This orthobiological approach is important due to the steep 48% increase in disease prevalence that has been reported over the past three decades (1990–2019) and is now considered a leading cause of disability in older adults. These data are concerning but probably underestimate the true size of the problem [49]. The progress of developing new treatments is also quite slow compared to the vast new theory surrounding this disease from degenerative, inflammatory, and genetic involvement. The global burden of knee arthroplasty also increases, and the number of revision procedures should not be forgotten. Comprehensive nationwide data from Germany shows that the annual incidence of TKA steadily increased by 32.4% from 2005 until 2018 and is projected to increase by up to 43% in the incidence rate of primary TKAs to 299 per 100,000 inhabitants in 2050. The annual total number of revision procedures is forecast to increase even more rapidly by almost 90% or 62.7 revisions over 100,000 people from 30.8 in 2018 [57]. Another study in the US also reported that TKA is expected to increase 69% by 2050 compared to 2012, from 429 procedures/100,000 in 2012 to 725 in 2050. This translates into a 143% projected increase in TKA volume [52]. In Australia, the rate of TKA was 123 per 100,000 population in 2003, and by 2013, the rate of TKA had increased to 213 per 100,000 population, costing approximately $AUD448 million, rising to $AUD905 million in 2013. This number is projected to increase up to 248 TKAs per 100,000 population, at an estimated cost of $AUD1.38 billion to the health care system [3]. 

Until now, orthobiologics have not been included as a current practice for knee OA. However, this approach is not meant to replace the current therapy of nonoperative or even operative treatment but to serve as an intermediate intervention. The operative treatment itself is prone to survivorship problems, chronic postoperative pain, and even infection. The idea of improving treatment before arthroplasty is paramount, and some examples of orthobiologic agents used in knee OA have been proven to have positive clinical results, mostly in early or moderate knee OA. Could orthobiologics be included in the consensus of therapy for knee OA, is it just a dream or reality?

## Blood-derived products

“Blood-derived products” is the term that is used to describe the variety of products from peripheral blood that are processed through different techniques. Some examples of blood-derived products are platelet-rich plasma (PRP), platelet-rich growth factors (PRGF), platelet-rich fibrin (PRF), autologous conditioned serum (ACS), alpha-2-macroglobulin (A2M), and several others. The most common blood-derived product that is used in orthobiologic treatment is PRP.

Platelet-rich plasma (PRP) is a solution of densely concentrated platelets with a minimum concentration of 3-5-fold from baseline that can be used to treat osteoarthritis [21]. It is created by harvesting autologous whole blood and centrifuging it to concentrate the platelets. This solution is rich in anti-inflammatory and anabolic proteins and has been shown to induce chondroprotection, leading to its use in the treatment of degenerative conditions such as OA. PRP is the most widely used orthobiologic because it is easily obtained and applied and is more accessible from both regulatory and operational perspectives [67], moreover, it has been established that PRP is most effective for earlier stages of OA [9, 58]. 

The diversity of PRP publications is related to the variability of platelet blood counts among people, along with the technical impossibility of analysing platelet concentrations in everyday practice; the variety of PRP types is the main factor responsible for the discrete quality of methodology in most studies. There is also a lack of consensus regarding the preparation of standardized doses with an appropriate absolute number of platelets and concentration [8]. 

Several studies demonstrated the superior effect of PRP compared to placebo [27, 39, 41, 58, 72, 82], although several studies did not show a significant difference in symptoms and joint structure [11, 26, 28] within 6–12 months.

Different classification systems have been proposed. For example, Ehrenfest et al. first classified PRP based on three main variables: platelet, leukocyte, and fibrin content, resulting in four main categories of PRP: pure PRP (P-PRP), leukocyte-rich PRP (LR-PRP), pure platelet-rich fibrin (P-PRF), and leukocyte-rich PRF. Then, PRP classifications were based on leukocyte content, for example, leukocyte-rich PRP (LR-PRP) and leukocyte-poor PRP (LP-PRP) [25, 34]. 

In 2021, the latest AAOS guidelines suggested that LR-PRP and LP-PRP treatments could have different effectiveness when used to treat knee OA. While the number of studies is limited and the choice between LR-PRP and LP-PRP is still inconclusive, at this time, AAOS appears to prefer LR-PRP treatment [2]. 

Several other varieties of PRP have been used, including photoactivated PRP (PA-PRP) and growth factor concentrate from PRP. The concept of a photoactivator in PRP is to improve inflammatory mediators [81, 100]. Therefore, the photoactivated process works synergistically in the activation of PRP. Although no difference was found between PA-PRP and HA, PA-PRP did improve the symptoms and functional outcome. However, some patients had minor reactions of pain and swelling after the injection of PA-PRP [73]. The growth factor concentrate from PRP was injected as an acellular growth factor-rich concentration. The first to use plasma rich in growth factors (PRGF) was Anitua et al., who reported improved symptoms and functional scores. PRGF is believed to improve symptoms and functional outcomes without the side effects of PRP [6, 76, 89]. 

In 2016, Gormelli conducted a randomized, double-blind trial of 162 patients with different stages of knee OA, who were randomly divided into four groups receiving 3 IA doses of PRP, one dose of PRP, and one dose of HA or saline injection, and each group was divided into early OA and advanced OA subgroups. There was a statistically significant improvement in the IKDC and EQ-VAS scores in all the treatment groups compared with the control group. The knee scores of patients treated with three PRP injections were significantly better than those of patients in the other groups. In the early OA subgroups, significantly better clinical results were achieved in the patients treated with three PRP injections, but there was no significant difference in the clinical results of patients with advanced OA among the treatment groups [40]. Although PRP treatment leads to improved clinical outcomes, PRP injection also has adverse reactions such as pain and swelling after injection. These adverse reactions are higher in LR-PRP than in LP-PRP [55]. 

The further question would be at what stage of knee OA does PRP yield better improvement. Most publications recommend PRP injection for the early stage of OA. Ismaiel in 2018 performed an RCT with sixty patients with grade III and IV knee OA treated with PRP or corticosteroids. The VAS score notably improved in both treatment groups at the 3- and 6-month follow-ups, although the variations were more significant in the PRP group [53]. In addition to using PRP in late-stage OA, the utility of PRP to postpone TKA has also been studied. Sanchez et al. evaluated the potential of PRP in postponing and even avoiding total knee replacement in patients with advanced knee OA. This study involved 186 patients who underwent TKA after PRP injections. Their analysis showed that the arthroplasty procedure was postponed for more than 1.5 years in 74.1% of patients, with a median of 5.3 years. Second, a survival analysis was conducted on 481 patients with grade III and IV OA receiving PRP injections. The analysis showed that 85.7% of the patients treated with PRP did not undergo TKA during the five-year follow-up. The severity of knee osteoarthritis did influence the delay to surgical intervention, and the survival rates were significantly higher in KL III patients than in KL IV patients [80]. Therefore, the effectiveness of PRP in KL IV is still in doubt clinically. PRP treatment in KL IV might be given in selected cases, such as inoperable patients due to comorbidities or patients who declined the surgery. However, one should remember to manage patient expectations before PRP treatment.

Although PRP has clinical benefits, cellular and magnetic resonance imaging (MRI) changes are scarce. Bellisari et al. found a reduction in T2 mapping in MRI evaluation of patellofemoral cartilage after the intraarticular injection of PRP [20]. Raeissadat et al. also found changes in patellofemoral cartilage volume and synovitis [77]. A different result was found by Halpern et al. [45] They found no changes in osteoarthritis exacerbation in the patellofemoral joint. The same result was also found by Buendía-López [17]. These different results might be due to the presence of leukocytes in some PRP preparations, PRP concentrations, dosages, follow-ups, and OA grades.

It is important to remember that knee osteoarthritis is often multifactorial. Mechanical problems such as malalignment might have a significant role in some cases, which can only be addressed with surgical approaches. Adding PRP might yield a superior outcome compared to realignment alone. Zhang, in his study, compared HTO only with HTO combined with PRP in patients with medial knee OA. The researchers found superior outcomes in the HTO combined with PRP group, not only in the medial joint space width but also in the Lysholm score and WOMAC [99]. Therefore, we also need to address the mechanical alignment problem (tibiofemoral malalignment and patellofemoral malalignment).

Although there is still no consensus regarding the optimal preparation, PRP concentration, dosage, follow-up, and OA grading in the treatment of knee osteoarthritis with PRP, no significant harm was found with this intervention compared to other treatments, such as hyaluronic acid, corticosteroids, and NSAIDs. PRP was proven to improve pain and function, but its cost-effectiveness compared with intra-articular injection of hyaluronic acid and corticosteroids has not yet been studied. Several insurance companies also have not approved the use of PRP. Therefore, to date, the use of PRP in knee osteoarthritis is common and accepted as a safe biologic treatment in orthopaedics. However, further research is needed regarding the efficacy of PRP not only in terms of structural, cellular, and molecular changes but also with respect to OA severity, newer formulations, biomaterials, combinations, and newer models of delivery.

Autologous conditioned serum (ACS) is rich in interleukin-1 receptor antagonist protein. Inflammation plays a key role in OA pathophysiology. Proinflammatory and matrix metalloproteinase (MMPs) are upregulated in tissue and synovial fluid [94], including the interleukin receptors on chondrocytes and synovial fibroblasts [79]. The interleukin-1 receptor antagonist, as a competitive receptor antagonist, can inhibit the effect of interleukin-1 [24]. It was used as a therapeutic agent and created by Meijer et al. as ACS [63]. ACS was proven to improve the functional score for knee OA but did not change the grade of knee OA [7, 18, 82, 97]. Sundman et al. studied the effects of ACS on the expression of anabolic and catabolic genes and the secretion of nociceptive and inflammatory mediators and compared it with HA. They found a significant decrease in catabolism and MMP-13 and an increase in hyaluronan synthase-2 and cartilage synthetic activity compared with HA. Therefore, ACS might stimulate endogenous HA production. ACS also suppresses inflammatory mediators and the expression of their genes in synoviocytes and cartilage [85]. 

Alpha-2 macroglobulin (A2M) is a serum protease inhibitor that inhibits cartilage oligomeric matrix protein-cleaving proteinases (comp), MMP-13 and proinflammatory cytokines (ΙΛ‑1 β and tumour necrosis factor‑α) [62, 96]. Therefore, A2M acts as a chondrogenic and chondroprotector. Animal studies showed a slower rate of OA progression [22, 93]. However, A2M only works in acute flares because these endoproteases are only increased in acute flares of OA. However, no human clinical study has been performed to evaluate A2M in knee OA.

## Mesenchymal stem cells (MSCs)

The biological potential of MSCs has the greatest potential for curing degenerative disease due to their self-renewal ability, stemness maintenance, and potential for differentiation into cells forming multiple mesodermal tissues (plasticity). They can migrate towards injured tissues (homing/trafficking), where they display trophic effects (synthesis of proliferative, proangiogenic, and regenerative molecules) [36]. The regenerative effects of MSCs are due to their structural contribution to tissue repair and their immunomodulatory and anti-inflammatory action through direct cell–cell interaction or secretion of bioactive factors [37]. 

However, due to the great ability of MSCs to differentiate into different tissues, there is a possibility of—in addition to cancer or immunological disease—the differentiation of these cells into unwanted tissue, as described by Breitbach et al. in 2007, who found the calcification of MSCs injected into infarcted rat hearts [14]. In the case of knee OA, targeted cartilage defect regeneration by MSCs could be disturbed by the formation of MSC-mediated endochondral ossification, thus jeopardizing the formation of good-quality tissue and the clinical outcome. This could happen in bone marrow and synovial sourced stem cells but less in adipose tissue stem cells at the expense of less chondrogenic potential [36]. In addition, MSCs are still not widely applied due to ethical issues in cell sources and expensive cell cultures.

Mesenchymal stem cells can be isolated from various sources in the human body, but bone marrow-derived mesenchymal stem cells (BMSCs), adipose-derived mesenchymal stem cells (ADMSCs) and synovial-derived mesenchymal stem cells (SDMCs) have recently become popular for the treatment of knee OA. MSCs derived from adipose tissue have been suggested to have the highest chondrogenic potential [29]. 

A meta-analysis in 2018 by Iijima with 35 studies of 2385 patients suggested that MSC treatment through intra-articular injection or arthroscopic implantation significantly improved knee pain, self-reported physical function, and cartilage quality. Minor adverse events (knee pain or swelling) were reported with a wide-ranging prevalence of 2–60%, with no severe adverse events occurring. However, the quality of evidence supporting this meta-analysis is considered to be low, suggesting that a better meta-analysis should be performed in the future [50]. 

Another meta-analysis from Qu in 2021 also showed the same results. All 9 high-quality RCTs involving 476 patients were included in this meta-analysis. The pooled estimate showed that the treatment of MSCs significantly reduced VAS, WOMAC pain, WOMAC stiffness, and WOMAC function scores over a long-term follow-up (12 or 24 months). However, regarding the IKDC and WOMAC total scores, MSCs also showed improvement in these outcomes, although this improvement was not statistically significant when compared to the control [75]. 

The administration of MSCs and the outcomes of such treatment are also related to the number of cells injected into the knee. A study from Gupta in 2016 showed that a trend towards improvement was seen in the 25-million-cell dose group for all subjective parameters (VAS, ICOAP, and WOMAC scores), although this improvement was not statistically significant compared to that with placebo. Adverse events of knee pain and swelling were predominant in the higher dose groups (50, 75, and 150 million cells). The possible reason for this problem was the limited intra-articular space of the knee joint; the dose of 25 × 10^6^ cells might be optimal, and because of the high cell concentration or limited space in the knee joint, doses higher than 25 × 10^6^ cells might result in cell aggregation, which subsequently causes cell death [43]. 

The fear of adverse events of unwanted tissue in MSC application is normal, considering the nature of the potential differentiation of stem cells. However, Wakitani et al. demonstrated the safety of using BMSCs in 41 patients over a long-term follow-up until 137 months after transplantation: neither tumours nor infections were observed. The debate is still ongoing and warrants close scrutiny since such stem cell therapies are far from being accepted in the field of clinical articular cartilage repair, nor has their long-term safety been convincingly proven [92]. 

A meta-analysis on the application of ADMSCs by Gadelkarim with 15 studies of 463 patients. The results were significant improvement in quality of life (QOL) among the three dose subgroups (high, medium, and low doses). However, after a year, the results were no longer significant, and the results of the double-arm analysis did not confirm the previous positive findings, which means that further larger and long-term follow-up is needed to support evidence on ADMSC application [38, 54]. 

Although MSC therapy showed promising efficacy in increasing joint function and reducing pain in knee OA patients, its wide application was hindered by regulatory and ethical issues and expensive cell culture costs. Another alternative cell-based regenerative therapy for knee OA is secretome therapy. Recent studies have suggested that the main therapeutic benefits of MSCs are not limited solely to their cell-to-cell interactions. MSCs secrete a broad range of bioactive molecules, including proteins, nucleic acids, proteasomes, exosomes, microRNAs and membrane vesicles, collectively known as the secretome. The stem cell secretome is a collective term for the paracrine soluble factors produced by stem cells and utilized for their intercell communication. In addition to intercell communication, paracrine factors are also responsible for tissue development, homeostasis and regeneration [4]. 

Three meta-analyses of in vivo animal studies showed that MSC secretomes were effective in promoting the repair and regeneration of osteochondral defects, resolving inflammation, and alleviating OA degeneration. In most studies, secretome-treated animals displayed increased cellular proliferation, enhanced matrix deposition, and improved histological scores. Based on the relevant preclinical animal studies reported to date, these systematic reviews show the therapeutic benefit of MSC secretome therapy in cartilage repair [13, 23]. One of the meta-analyses also suggests that both stem cells and secretome interventions show similar effects in improving cartilage regeneration in animal trials [64]. 

Combining the secretome with other regenerative modalities, such as MSCs and PRP, can also result in better formation of glycosaminoglycans and collagen II contents and articular cartilage preservation, as shown in a recent study by Nabavizadeh et al. These combinations also showed the lowest expression of MMP3 and the highest expression of SOX9 protein. Injecting a combination of MSCs/secretomes/PRP can result in better efficacy in terms of joint space width, articular cartilage surface continuity and integrity, subchondral bone and ECM constituents [65]. 

Although there are limited reports of the negative effects of the secretome, there are always potential risks using exogenous biological molecules, although these risks are reduced when compared to cell-based therapies. A comprehensive analysis is needed before secretome administration to specific niches in different tissues. Secretomes containing exosomes and extracellular vesicles can be immunogenic, but they appear to be less immunogenic than their parent MSCs [98]. However, some adverse events might occur with MSC-based therapy, including transient arthralgia, swelling of the joint after local injection [74], oedema and cramps [83], and the most severe event is angina pectoris in patients with hypertension and hyperlipidaemia [74]. However, several aspects should be clarified, including the suitable cell source, production protocol, choice of suitable patient for this treatment, and choice of the right conditioned media to minimize adverse events.

From all of the RCTs and meta-analyses described above, those results should be interpreted carefully because each study has variably different methods for applying MSC-based therapy for knee OA. There are no standardized consensus views or guidelines in MSC-based therapy among these studies even today. There are still major questions that need to be answered by further research on several topics, such as what is the optimal source of MSC-based therapy (bone marrow, adipose, synovial, umbilical cord), number of cells to be injected, production protocol and method of administration (intra-articular injection, arthroscopic implantation, combined with mashed cartilage or scaffold), dosing, interval, duration of injection, follow-up, choice of suitable patients for this treatment, and choice of the right conditioned media to minimize adverse events. These technical aspects need to be standardized to be safely and effectively applied in daily clinical practice and to yield replicable results by other practitioners.

## Gene therapy

OA is a multifactorial disease, and one of those factors is genetic. Some studies have shown several genes that are correlated with the osteoarthritic process [5, 16, 84, 88, 87, 90, 15, 35, 47, 56, 59–61, 78]. The understanding of osteoarthritis as an ongoing inflammatory disease confirms the urgent need for treatment with sustained benefits. With those problems, gene therapy might not only serve to control the genetic problem but also yield long-term therapeutic effects in protecting and rebuilding the articular cartilage. Therefore, we need treatment options that can maintain the therapeutic concentration in a prolonged and regulated manner. With the anatomy and biomolecular nature of the joint, it is difficult to deliver drugs to joints sustainably, and such treatment requires repeated systemic introductions. However, daily injection fails to maintain therapeutic serum levels [10]. 

Gene therapy was introduced to enable patients to synthesize endogenous proteins. The gene therapy was injected intra-articularly to minimize systemic adverse events and due to the anatomy and biomolecular properties of the joints. Materials were more likely to escape in the first week after being injected [31, 33]. Small molecules escape through synovial capillaries, while macromolecules escape through the lymphatics. Therefore, although it is safer to perform intra-articular gene therapy injection, there is always a possibility of delayed adverse events due to the genetically modified cells that migrate extra-articularly.

The concern regarding gene therapy in osteoarthritis is also focused on the vector that is used. Some studies used retrovirus vectors for ex vivo gene therapy, and some studies used adeno-associated virus (AAV) for in vivo gene therapy. AAV is used more often because it can penetrate deeply within articular cartilage and transduce chondrocytes in situ. The ex vivo protocols also have issues. Some cells injected intra-articularly are prone to escape from the joint. The mesenchymal stem cells (MSCs) that are injected intra-articularly are cleared rapidly from the joints [48, 68]. However, this approach is controversial, and for this reason, in vivo gene therapy is favoured. AAV has become a good vector for in vivo gene therapy. Some studies showed that transgene expression can be regulated to match disease activity with AAV in vivo gene therapy [69, 70, 86]. However, acquired immunity may be an issue since some populations have neutralizing antibodies to AAV serotype 2, which can present in synovial fluid as well as in serum and is less likely for AAV serotype 5 [12]. Even so, the effect of AAV after intra-articular injection can be augmented by using empty AAV capsids as decoys for synovial macrophage function [1]. Another concern regarding the administration of in vivo gene therapy intra-articularly is synovial cell turnover. A previous study showed that only 25% of the early level persisted in the joint for the rest of the animal’s life [42]. However, this outcome is different in every animal and based on age [95]. 

Evans et al. investigated gene transfer to human joints in rheumatoid arthritis (RA). They studied 9 patients with RA with intraarticular MCP joint injection of IL-1 receptor antagonist (IL-1Ra) cDNA retrovirus-mediated. They found high expression levels of IL-1Ra in the synovium of the injected joints with adverse events [32]. With the anti-inflammatory and anti-erosive properties of recombinant IL-1Ra, it was expected that a high level of IL-Ra would improve RA. This is also expected in OA.

Nonviral vectors are also used in OA treatment. However, this results in insufficient levels and durations of gene expression. Such nonviral gene transfer was used to induce chondrogenesis in MSCs. A scaffold system was employed for transcription factor gene delivery by enhanced chondrogenesis of adipose stem cells on porous polylactide-co-glycolide (PGLA) containing plasmid DNA encoding SOX [51] or MSCs [71]. 

Preliminary studies targeting transforming growth factor beta (TGF‑β1) expression showed improvements in pain, function, and physical ability. This showed a promising treatment for cartilage degeneration [19, 44]. Other studies of gene therapy have also been performed on the IL-1 pathway. A clinical study is underway to focus on IL1Ra gene therapy in knee OA [30]. 

Choosing the transgene in OA gene therapy is an option. Undergoing study in gene therapy is now focusing on tumour transforming growth factor (TGF)-β [44], insulin-like growth factor-1, interleukin-10 (IL-10) [46, 66], and interferon (IFN)-β.

Gene therapy is a strategy that focuses on specific targets in OA and is expected to have long-term therapeutic effects involving structural changes and to alleviate symptoms in early OA. The concept of gene-based therapy requires safe and targeted strategies. Further research that focuses on controlling gene expression in OA will provide clinical benefits.

## Conclusion

Orthobiologic therapy is a promising modality to be included in the armamentarium of therapy for knee OA. Even though only PRP treatment is considered realizable rather than just a dream, MST and its derivatives should be continuously and thoroughly investigated. Publications on PRP for knee OA have dominantly reported improvements in earlier stages of OA, and the effect of this treatment surpasses the placebo effect after 6 months of therapy. The application of PRP for late-stage OA still leads to improvement and could postpone TKA. However, patient selection is still important because some studies have shown that higher BMI, older age, and later stages of OA may decrease the benefit of PRP therapy. Standardization of the PRP preparation should be globally discussed to improve the quality of the studies and patient outcomes.

For MSC application, the current literature showed generally the same results for MSC application as for PRP treatment, with dominant significant improvement in pain and functional outcomes after 3–6 months that lasted up to 12 months. However, a large sample of high-quality publications to support this evidence is still lacking. As the cost of MSC therapy is much higher than that of PRP, the former might not be suitable for wide application for knee OA globally.

Treating knee OA as a genetic disease might provide a long-term therapeutic effect of structural changes and alleviate symptoms in early OA. However, this cannot be used globally as an option for treating knee OA before further studies prove the clinical benefits in multicentre and level 1 studies.

One of the authors, N.C.B., receives payment or benefits from DePuy Johnson & Johnson, and Zimmer Biomet, and sits on the editorial board of CORR, OJSM, Journal of Orthopaedic Surgery.
